# Unravelling the cardio-renal-metabolic-foot connection in people with diabetes-related foot ulceration: a narrative review

**DOI:** 10.1186/s12933-024-02527-1

**Published:** 2024-12-18

**Authors:** Nick S. R. Lan, Girish Dwivedi, P. Gerry Fegan, Fran Game, Emma J. Hamilton

**Affiliations:** 1 Centre of Excellence for Cardiometabolic Health, Fiona Stanley Hospital, Perth, Australia; 2 Department of Cardiology, Fiona Stanley Hospital, Perth, Australia; 3 Medical School, The University of Western Australia, Perth, Australia; 4 Harry Perkins Institute of Medical Research, Perth, Australia; 5 Medical School, Curtin University, Perth, Australia; 6 Department of Endocrinology and Diabetes, Fiona Stanley Hospital, Perth, Australia; 7 Department of Diabetes and Endocrinology, University Hospitals of Derby and Burton NHS Foundation Trust, Derby, UK; 8 Centre of Excellence Multidisciplinary Diabetes Foot Ulcer Service, Fiona Stanley and Fremantle Hospitals Group, 11 Robin Warren Drive, Murdoch, Perth, Australia

**Keywords:** Cardiovascular diseases, Coronary artery disease, Diabetes, Foot ulcer, Heart failure, Risk factors

## Abstract

Diabetes-related foot ulceration (DFU), a serious but preventable complication of diabetes, is a leading cause of hospitalisation, lower extremity amputation and disability worldwide. People with DFU have a greater burden of cardiovascular risk factors, heart failure and chronic kidney disease, resulting in over two-fold higher risk of cardiovascular death compared with people with diabetes without DFU. Here, we propose a “cardio-renal-metabolic-foot” connection in people with diabetes based on shared pathophysiological mechanisms linking DFU with cardiovascular and renal disease. Whilst these mechanistic links remain to be fully elucidated, systemic inflammation and infection in the context of DFU are postulated as key mediators in the development, and progression of, cardiovascular and renal disease. However, cardiovascular and renal disease are also implicated in the pathogenesis of DFU, highlighting the multi-directional interplay between conditions. The impact of screening, prevention, and early management of cardiovascular complications associated with DFU requires further research. Multi-modality cardiac imaging could play a role in unravelling disease mechanisms leading to novel therapeutic strategies, as well as facilitating personalised risk assessment and management. Recent clinical trials have transformed the therapeutic landscape for people with type 2 diabetes, by demonstrating that sodium glucose co-transporter 2 inhibitors, glucagon-like peptide-1 agonists and non-steroidal mineralocorticoid receptor antagonists improve cardiovascular and renal outcomes. Although dedicated research in people with DFU is warranted, these therapies could target multiple facets of the “cardio-renal-metabolic-foot” connection. The holistic, person-centred approach to managing DFU should incorporate new multidisciplinary models of care focusing on the prevention and management of cardiovascular and kidney disease.

**Graphical abstract:**

The cardio-renal-metabolic-foot connection in people with diabetes. There is a critical need for (1) a better understanding of mechanisms connecting DFU with cardiovascular and kidney disease, perhaps guided by cardiac imaging, novel biomarkers, multi-omics and artificial intelligence to facilitate current treatments and the development of novel therapeutic strategies, (2) more data from clinical trials, registries and biobanks to inform clinical guidelines and evidence-based medicine, and (3) health system-wide integration of structured models of care with a contemporary emphasis on cardio-renal-metabolic-foot health to improve patient outcomes. *DFU* diabetes-related foot ulceration. *Created with BioRender.com*
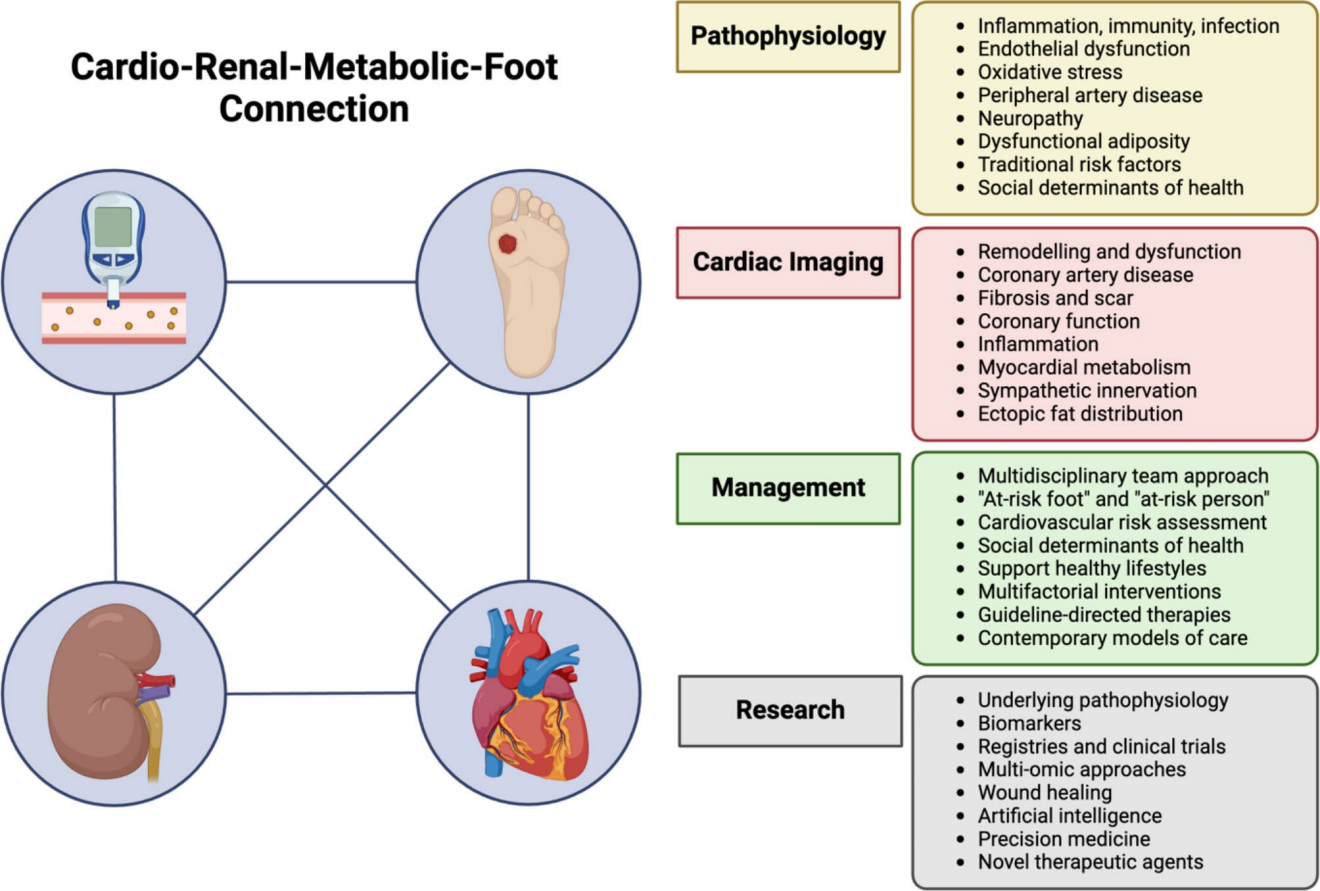

## Introduction

Diabetes mellitus increases the risk of cardiovascular (CV) death by over two-fold and is a leading cause of morbidity and mortality worldwide [1, 2]. Over half a billion people are living with diabetes and this number is projected to more than double by 2050 [2]. Diabetes is a cardiometabolic disorder associated with an increased risk of atherosclerotic cardiovascular disease (CVD), heart failure, chronic kidney disease (CKD), and other comorbidities [3]. A multifactorial strategy to reduce CV risk and the complications of diabetes, is therefore, an essential component of diabetes care [4, 5]. Microvascular disease (neuropathy, nephropathy, and retinopathy), younger age of onset of type 1, and a diagnosis of type 2 diabetes at ≤40 years age are associated with significantly greater risks of developing atherosclerotic CVD and heart failure [6–9]. Diabetes-related foot ulceration (DFU) is a highly morbid, yet preventable, complication of diabetes that is caused by peripheral neuropathy and/or peripheral artery disease (PAD), both of which are independently associated with increased CV risk [10]. 

Among people with diabetes, the annual incidence of DFU is 2–6%, with a lifetime incidence of up to 34% [11, 12]. In 2016, it was estimated that 131 million people (1.8% of the global population) were affected by diabetes-related lower extremity complications, resulting in 16.8 million years lived with disability [13]. DFU is a leading cause of hospitalisation and lower extremity amputations, resulting in reduced quality of life and high healthcare costs [14, 15]. Crucially, 5-year mortality associated with DFU is 50%; this is higher than the 5-year pooled mortality of 31% for all reported cancers [16–18]. Despite being a preventable condition, coronary artery disease (CAD) remains the main cause of death (30–60% of deaths) in people with DFU [17, 19–21]. Moreover, CKD affects 40–60% of people with DFU and there is a well-defined bi-directional association between renal and cardiac dysfunction, termed the cardio-renal syndrome [21–23]. This highlights the need for research and novel models of care that addresses what we propose as the multi-directional “cardio-renal-metabolic-foot” connection (*graphical abstract*) in people with diabetes.

This narrative review aims to outline the existing understanding of the association between DFU and cardiac disease, to explore the role of cardiac imaging, to propose a novel model of care addressing the cardio-renal-metabolic-foot connection, and to suggest avenues for future clinical research.

## Epidemiological findings

In the ADVANCE-ON study of 11,140 people with type 2 diabetes at high CV risk, the risk of incident death and CV events was significantly higher in the 300 (2.7%) people with a history of lower extremity ulceration or amputation (composite variable), even after adjusting for potential confounders [24]. People with a history of lower extremity ulceration or amputation had a 1.5-fold higher risk of non-fatal myocardial infarction compared to those without lower extremity ulceration, amputation or peripheral revascularization [24]. Moreover, a history of lower extremity ulceration or amputation conferred similar risk of death and CV events as a history of peripheral revascularisation [24]. Although these observational data highlight the relatively poorer survival and CV outcomes in people with type 2 diabetes and a history of major PAD, they do not answer the specific question as to whether a history of DFU increases CV risk. On the other hand, a propensity score-matched retrospective population study showed that the risk of incident CV events in people with type 2 diabetes and diabetes-related foot disease (which includes DFU) is more than double that of people with type 2 diabetes without diabetes-related foot disease [25]. The study suggests that diabetes-related foot disease is intrinsically associated with poorer outcomes beyond the classical contributing factors, such as other CV risk factors, microvascular disease and macrovascular disease [25]. Notably, another study of 2880 people with neuropathic DFU, who did not have significant PAD, demonstrated that CAD was still the most frequent cause of death [21]. Whilst predictors of CV death were not reported in the aforementioned study, nephropathy and lower extremity amputation were strong predictors of all-cause death in people with DFU [21]. 

A recent systematic review and meta-analysis demonstrated that people with DFU had a significantly higher incidence of ischaemic heart disease and stroke, and an over two-fold increased risk of CV death compared with people with diabetes without DFU [26]. Whilst the incidence of heart failure was higher in people with DFU, this was not statistically significant [26]. Reflecting the lack of literature, the authors found only 5 observational studies (6788 people from 5 countries) that directly compared CV-related morbidity between those with DFU versus those with diabetes but without DFU [26]. Large cohort studies, including a study of 414,523 people with diabetes (20,737 [5.0%] with DFU), have demonstrated that DFU is independently associated with all-cause death [16, 22, 27]. Moreover, DFU-specific factors such as chronicity, severity, location, infection, amputation, and recurrence are associated with a higher risk of death; yet, studies relating these factors to CV events are lacking [28–34]. It is difficult to ascertain whether DFU per se increases the risk of CV events or whether it is a marker of “medical frailty”. People with DFU tend to have a higher prevalence of CV risk factors, heart failure, CKD, atherosclerotic CVD (e.g., PAD) and confounders (e.g., lifestyle, socioeconomic disadvantage or genetic predisposition) that can increase the risk of CV events but which cannot be fully accounted for in observational studies [16, 22, 35, 36].

## Pathophysiological link

**Fig. 1:**
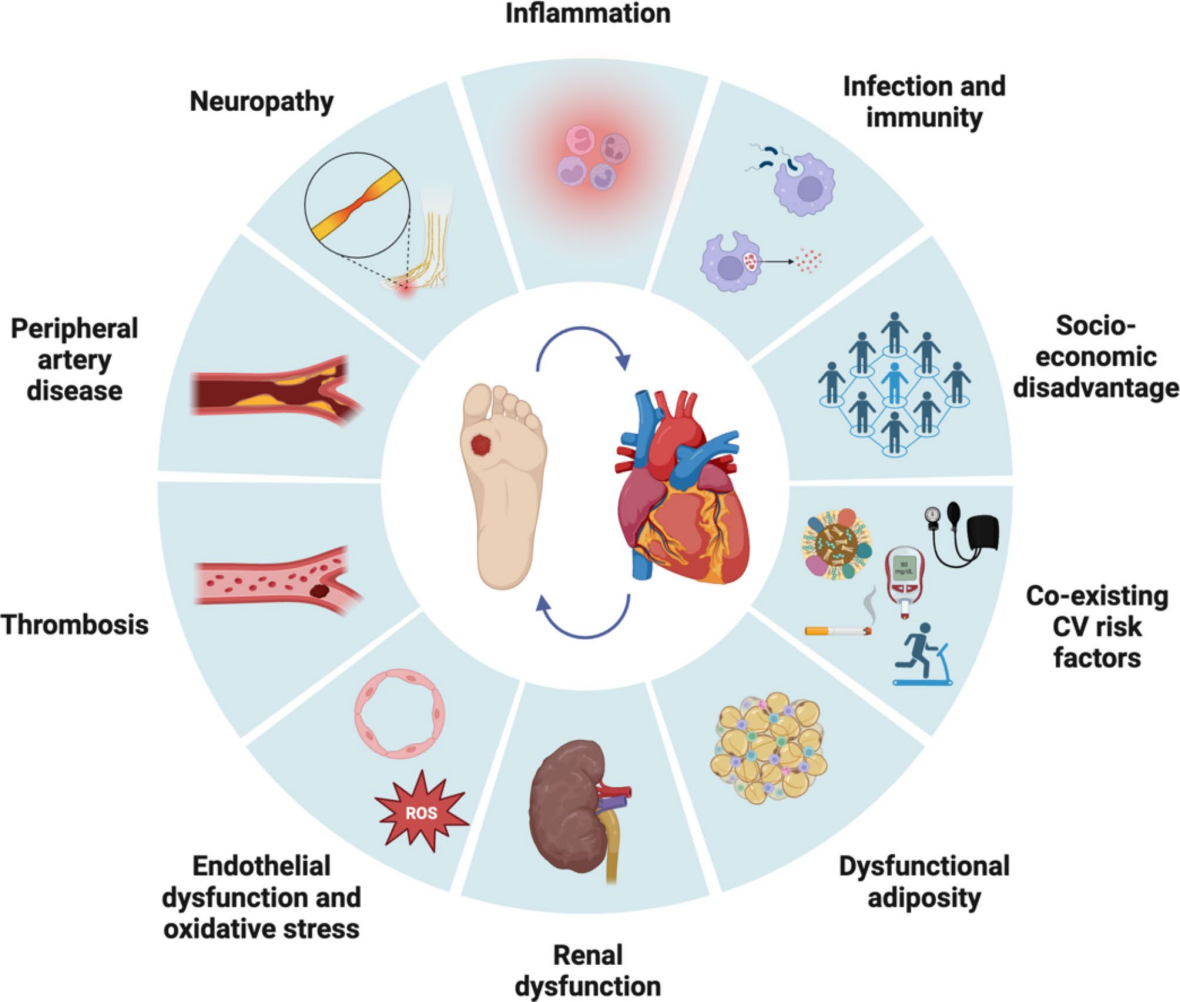
Proposed mechanisms connecting DFU with cardiac disease. The complex link between DFU and cardiac disease is poorly defined but is likely to be multifactorial and bidirectional. Inflammation, endothelial dysfunction, and oxidative stress may contribute to accelerated atherosclerosis, thrombosis, and cardiomyocyte dysfunction in the setting of DFU. People with DFU are often at socioeconomic disadvantage and have concomitant CV risk factors, renal dysfunction, neuropathy, and peripheral arterial disease that further increases the risk of CV complications. *CV* cardiovascular, *DFU* diabetes-related foot ulceration, *ROS* reactive oxygen species. *Created with BioRender.com*

A DFU is defined as a break in the skin of the foot that involves as a minimum the epidermis and part of the dermis, and occurs below the malleoli in a person with diabetes [37]. DFU usually develops following a precipitating event (including minor trauma) in a person with risk factors for DFU, these include: peripheral neuropathy, PAD, foot deformity, prior DFU, prior lower extremity amputation and end-stage renal failure [38, 39]. Peripheral neuropathy can result in the loss of protective sensation and sometimes foot deformity; the combination of reduced protective sensation, foot deformity and limited joint mobility leads to abnormal biomechanical loading of the foot [38]. The high mechanical stress in certain areas of the foot results in callus formation (thickened skin), further increasing abnormal loading of the foot which eventually results in subcutaneous haemorrhage and skin ulceration [38]. Continued walking impairs healing of the ulcer in the insensitive foot, underscoring the need for offloading [38]. The majority of ulcers are purely neuropathic (35%) or neuro-ischaemic (50%), with a small percent being purely ischaemic (15%) [40]. Several classification systems have been validated to assess and stage DFU including the SINBAD system, WIfI classification and IDSA/IWGDF infection classification systems [40–43]. DFU and cardiac disease share overlapping pathophysiological mechanisms (Fig. 1) and it is possible that a bidirectional relationship exists, with each condition potentially worsening the other [44]. 

### Neuropathy

Neuropathy is a microvascular complication resulting from chronic hyperglycaemia, metabolic dysfunction and inflammation that affects sensory, motor, and/or autonomic nerve fibres. Given that microvessels form neurovascular networks with accompanying nerves, neuropathy in diabetes is often associated with endothelial dysfunction and consequently, accelerated atherosclerotic disease [45]. Neovascularisation arising from the vaso vasorum due to intimal hypoxia and ischaemia may also link microvascular disease and atherosclerosis [46]. Cardiac autonomic neuropathy, which is characterised by an imbalance between sympathetic and parasympathetic activity, can be present in 43–66% of people with DFU [47, 48]. Cardiac autonomic neuropathy manifests clinically as orthostatic hypotension, tachycardia, arrhythmias, left ventricular dysfunction, and silent myocardial infarction [47, 48]. Peripheral neuropathy and cardiac autonomic neuropathy are significant predictors of CV events; indeed, the presence of neuropathy is considered a diabetes-specific CV risk enhancer in the American College of Cardiology and American Heart Association guidelines [49–53]. However, people with diabetes, neuropathy and DFU may have a higher risk of cardiac disease than those with diabetes and neuropathy without DFU due to other potential mechanisms (see Fig. 1) [48]. 

### Peripheral arterial disease

PAD is a contributing factor in 50–70% of people with DFU and portends higher risk of impaired healing of the ulcer, gangrene, and lower extremity amputation [38, 40]. Crucially, PAD frequently co-exists with CAD. In people with neuro-ischaemic DFU, significant CAD has been observed in around 60% [54]. Although there are differences in the pathological basis of PAD and CAD, both conditions occur as manifestations of the systemic atherosclerotic process with shared CV risk factors such as smoking, hypertension, hypercholesterolaemia, diabetes and renal dysfunction [55, 56]. Indeed, people with PAD tend to also harbor more extensive, calcified and progressive CAD [57]. The co-existence of PAD and CAD (i.e., polyvascular disease) confers even higher risk of CV events and death than either alone, highlighting the extreme CV risk faced by many people with DFU [56–58]. In people with diabetes, PAD tends to differentially affect distal arteries and is characterised by medial arterial calcification, leading to arterial wall stiffness, as opposed to intraluminal atherosclerosis in people with PAD without diabetes [59]. A study has shown that below-the-ankle arterial disease may be a better predictor of CAD in people with DFU compared with above-the-ankle arterial disease [54]. 

### Inflammation

Inflammation, endothelial dysfunction and oxidative stress associated with DFU may be crucial factors that drive the development and progression of CAD and heart failure, as well as CKD [60–62]. The presence of DFU induces a systemic inflammatory cascade through an increased expression of cytokines and acute-phase proteins (e.g., C-reactive protein, interleukin-6, interleukin 1-beta, fibrinogen and tumour necrosis factor alpha) that correlates with ulcer severity [63, 64]. Heightened inflammation from DFU may accelerate atherosclerosis, result in a pro-coagulable state, and cause plaque rupture and myocardial infarction [65]. It has been recently hypothesised that inflammation due to repeated surgical insults such as debridement and/or amputation, surgery-related complications (e.g., blood loss or infection) and anaesthesia-related complications (e.g., haemodynamic instability or dehydration) can further stress the CV system in people with DFU [26]. Further research is required to confirm this hypothesis, however inflammatory biomarkers after amputation have been shown to predict death in people with DFU [33]. Metabolic alterations such as insulin resistance, hypertension, and atherogenic dyslipidaemia (e.g., oxidised low-density lipoprotein particles, dysfunctional high-density lipoprotein particles and elevated triglyceride-rich lipoproteins) further perpetuates atherosclerosis [10]. People with DFU have higher interleukin-6 and resistin levels, higher rates of microalbuminuria, and lower adiponectin levels compared with people with diabetes without DFU; this has led to an hypothesis that an “adipo-vascular axis” contributes to CV risk [66]. Notably, excess and dysfunctional adiposity (particularly visceral adiposity and other ectopic fat deposition) results in insulin resistance, systemic inflammation and oxidative stress, and is central in the development of cardiovascular-kidney-metabolic syndrome [3]. 

### Immunity and infection

The immune system is implicated in atherosclerosis and in neuropathic DFU [60, 67, 68]. In terms of atherosclerosis, the low-grade inflammatory response attracts cells of the innate (e.g., monocytes and macrophages) and adaptive immune systems into atherosclerotic plaque [60, 67]. Importantly, over half of DFUs become acutely infected, manifesting as soft-tissue, bone (i.e., osteomyelitis) and/or systemic infection [10]. Superimposed infection of DFU may be polymicrobial and is typically associated with pronounced inflammation and immune system activation and dysfunction. Also, infecting microorganisms can mediate inflammation directly through infection and activation of vascular cells or indirectly through induction of a systemic immune response [60, 67]. Intriguingly, bloodstream infection with *Staphylococcus aureus*, a common infecting organism in people with DFU, is associated with increased risk of myocardial infarction [69, 70]. It is increasingly recognised that acute infections may trigger myocardial infarction, potentially due to the cytokine response causing plaque rupture [70, 71]. Here, a recent study has shown that deep and/or infected DFUs may be associated with an increased risk of incident CV events compared with DFUs that are not deep or infected [72]. 

### Chronic kidney disease

Nephropathy in the setting of diabetes is closely related to other microvascular complications such as neuropathy, macrovascular complications such as CAD and PAD, and heart failure [3]. CKD is a recognised risk factor for the development and recurrence of DFU, and is a significant predictor of adverse DFU outcomes such as poor healing, amputation and death [62, 73–76]. People with end-stage renal failure receiving dialysis are at particularly high risk, with factors such as nutritional deficiency, uraemia, anaemia (leading to reduced tissue oxygenation), peripheral oedema, reduced tissue oxygenation during dialysis, and vascular calcification being postulated as mediators of either worsening neuropathy, poor DFU healing, or both [62, 77]. Medial arterial calcification, a disorder distinct from atherosclerosis, can occur in people with diabetes or chronic kidney disease, and is associated with development of DFU and worse CV outcomes [78]. It has been hypothesised that a bidirectional relationship exists between CKD and DFU, but ongoing studies are needed to confirm this potential connection [62, 77]. Chronic systemic inflammation due to DFU might contribute to the development and progression of CKD [62, 77]. Systemic inflammation, recurrent infection, and use of antibiotics might result in episodic acute kidney injury, further increasing the risk of CKD in DFU [77, 79]. The development and progression of CKD perpetuates worse DFU outcomes and amplifies the risk of atherosclerotic CVD and heart failure.

## Role of non-invasive cardiac imaging

Cardiac investigations, including electrocardiography and natriuretic peptides, should be performed where appropriate when cardiac disease is suspected in people with DFU. Non-invasive cardiac imaging (Table 1) could add incremental value by (1) facilitating the use of preventive or prognostic therapies through earlier detection of CAD and myocardial disease, (2) monitoring disease progression or the impact of treatments, (3) personalising risk assessment and prognostication, and (4) unravelling disease mechanisms that will inform research into novel therapeutics and precision medicine for people with DFU. Cardiac imaging would supplement the findings from electrocardiography, an inexpensive and relatively easily accessible bedside test. Studies have suggested that QTc prolongation predicts all-cause and cardiac mortality in people with DFU [80, 81]. QTc prolongation has also been associated with severe peripheral arterial disease in people with DFU [82]. Moreover, signals of potential mortality benefit were seen in new attendees of multidisciplinary diabetes foot services who had an electrocardiogram (where 1 in 2 were abnormal) followed by appropriate clinical action [83]. 

**Table 1:** Multi-modality cardiac imaging for assessing cardiomyopathic processes and coronary artery disease associated with DFU [84, 99]

Process	Echocardiography	CCTA	CMR	SPECT/PET
Cardiac remodelling	LV mass, wall thickness and volume (including 3D)	LV mass, wall thickness and volume	LV mass, wall thickness and volume	LV mass, wall thickness and volume
Systolic function	LVEF (including 3D), tissue Doppler (s’) and speckle tracking (e.g., LV strain)	–	LVEF and strain (e.g., LV strain) from cine imaging	LVEF
Diastolic function	LA size (including 3D), pulse wave Doppler (E/A, E-deceleration), tissue Doppler (E/e’) and speckle tracking (e.g., LA strain)	–	LA volumes, mitral inflow and flow propagation, and strain (e.g., LA strain)	LV filling parameters (e.g., peak filling rate)
Coronary artery disease	–	Plaque burden, distribution, luminal stenosis, calcification, and highrisk plaque features^a^	Using coronary magnetic resonance angiography	–
Ischaemia and viability	Stress echocardiography	CT-derived fractional flow reserve and CT-perfusion	LGE, and perfusion defects before and after stress	Perfusion defects before and after stress
Fibrosis	–	–	LGE, T1 mapping and ECV fraction	–
Coronary function	Doppler-derived coronary flow velocity reserve	–	Myocardial blood flow	Myocardial blood flow
Peri-vascular inflammation	–	Peri-coronary adipose tissue attenuation	–	–
Myocardial metabolism	–	–	Magnetic resonance spectroscopy	PET using certain radiotracers
Sympathetic innervation	–	–	–	PET/SPECT using certain radiotracers
Ectopic fat	Epicardial adipose tissue thickness	Epicardial adipose tissue volume	Epicardial adipose tissue volume and composition	Epicardial adipose tissue volume and tracer uptake

*3D* 3-dimensional, *CCTA* coronary computed tomography angiography, *CMR* cardiac magnetic resonance, *CT* computed tomography, *DFU* diabetes-related foot ulceration, *ECV* extracellular volume, *LGE* late gadolinium enhancement, *LV* left ventricular, *LVEF* left ventricular ejection fraction, *PET* positron emission tomography, *SPECT* single-photon emission computed tomography

^a^Highrisk plaque features include low-attenuation plaque, positive remodelling, spotty calcification, and the napkin ring sign

### Echocardiography

Echocardiography could be a valuable tool in people with DFU, given that it is a commonly available modality with no radiation exposure, rates of abnormalities are likely to be high, and medical therapies to reduce the risk of heart failure are available. In the absence of ischaemic or congenital heart disease, hypertension, or significant valvular dysfunction, the finding of increased left ventricular mass, reduced left ventricular systolic function and/or impaired diastolic function suggests “diabetic cardiomyopathy”, which is associated with worse outcomes [84]. Echocardiographic studies have shown high rates of abnormalities in people with DFU, even in those without known cardiac disease or hypertension. In a study of 80 people with DFU, over 75% had left ventricular hypertrophy, systolic dysfunction and/or diastolic dysfunction [85]. Moreover, subclinical cardiac dysfunction may be more common in people with DFU compared with asymptomatic people with diabetes, where abnormal left ventricular strain is seen in almost 25% [86]. Intriguingly, an observational study of 54 people with DFU found that left ventricular global longitudinal strain was significantly lower in those with DFU, and that treatment of DFU was associated with improved strain [87]. The finding of improved left ventricular global longitudinal strain after DFU treatment was corroborated by another observational study of 62 people with DFU [88]. Echocardiography may uncover prior myocardial infarction, which would warrant intensive secondary prevention of CVD and potentially further investigations. The utility of routine echocardiography and its impact on management, outcomes, and cost-effectiveness in people with DFU should be evaluated.

### Cardiac computed tomography

Compared with people without diabetes, those with diabetes have more extensive and rapidly progressive CAD. Whilst the coronary artery calcium score improves the prediction of coronary heart disease events, routine screening for CAD in asymptomatic people with diabetes is not recommended by the European Society of Cardiology or American Diabetes Association guidelines [89–91]. However, people with DFU have a higher prevalence of CV risk factors and subclinical CVD than people with diabetes without DFU; thus, screening for CAD may be of greater value to guide CV prevention, facilitate early diagnosis and increase eligibility for risk-reducing therapies, some which may be costly and reserved for the highest risk [35]. Coronary computed tomography angiography (CCTA) provides a comprehensive assessment of coronary anatomy and extent of atherosclerosis with high resolution and low radiation (usually < 5 mSv) [92, 93]. Owing to technological advances, plaque quantification, composition and morphology can be accurately determined [92]. High risk plaque features (low-attenuation, positive remodelling, spotty calcification and “napkin ring” sign) and peri-vascular fat attenuation index, a marker of peri-coronary inflammation, can identify people at higher risk of myocardial infarction [94–96]. Peri-coronary inflammation may also be reduced by anti-inflammatory therapies [97]. There is therefore a need to determine whether DFU is associated with high risk plaque and peri-coronary inflammation. However, the applicability of CCTA in people with DFU may be reduced given the risk of contrast-induced nephrotoxicity. Photon counting detectors, CT-derived fractional flow reserve, CT-perfusion and artificial intelligence will further strengthen the utility of CCTA [92, 93, 98]. In the era of precision medicine, risk assessment incorporating both electrocardiography and imaging may offer a more comprehensive approach [83]. 

### Cardiac magnetic resonance

Whilst cardiac magnetic resonance imaging plays a crucial role in the diagnosis and management of several cardiac diseases, its role in the evaluation of diabetic cardiomyopathic processes as part of routine care is not well-established [99]. There is a complex interplay of myocardial metabolic remodelling that results in mitochondrial dysfunction, inflammation, and fibrosis in people with diabetes that could be exacerbated in the setting of DFU [99]. Using late gadolinium enhancement and T1 mapping methods, including extracellular volume fraction, cardiac magnetic resonance is able to assess replacement fibrosis (which could be due to unrecognised CAD) and diffuse interstitial fibrosis, both of which are associated with increased CV risk [100, 101]. In addition, T2-weighted imaging can assess tissue inflammation and magnetic resonance spectroscopy can measure cardiac metabolic processes such as oxygen consumption and short-chain fatty acid metabolism [99, 102]. 

### Cardiac nuclear imaging

Positron emission tomography can detect and quantify cardiac metabolic and molecular processes such as oxygen consumption, glucose metabolism, long-chain fatty acid metabolism, ketone body metabolism, lactate metabolism, inflammation and oxidative stress using certain radiotracers designed for targeting specific pathways [99]. Using certain radiotracers, positron emission tomography and single-photon emission computed tomography can detect coronary vasodilator dysfunction and cardiac autonomic neuropathy, both of which are associated with increased CV risk in people with diabetes [47, 48, 103, 104]. Cardiac single-photon emission computed tomography also provides information on left ventricular volumes, ejection fraction, and filling parameters, such as peak filling rate and time to peak filling rate, as well as myocardial perfusion [84]. However, cardiac magnetic resonance and nuclear imaging modalities may be best utilised in mechanistic studies examining the link between DFU and the heart, as they are more costly, may involve complex protocols, and are not as accessible or versatile as echocardiography [84]. Here, novel imaging biomarkers could enhance discovery and development of novel therapeutics in people with DFU [99, 104]. 

## Management implications

**Fig. 2:**
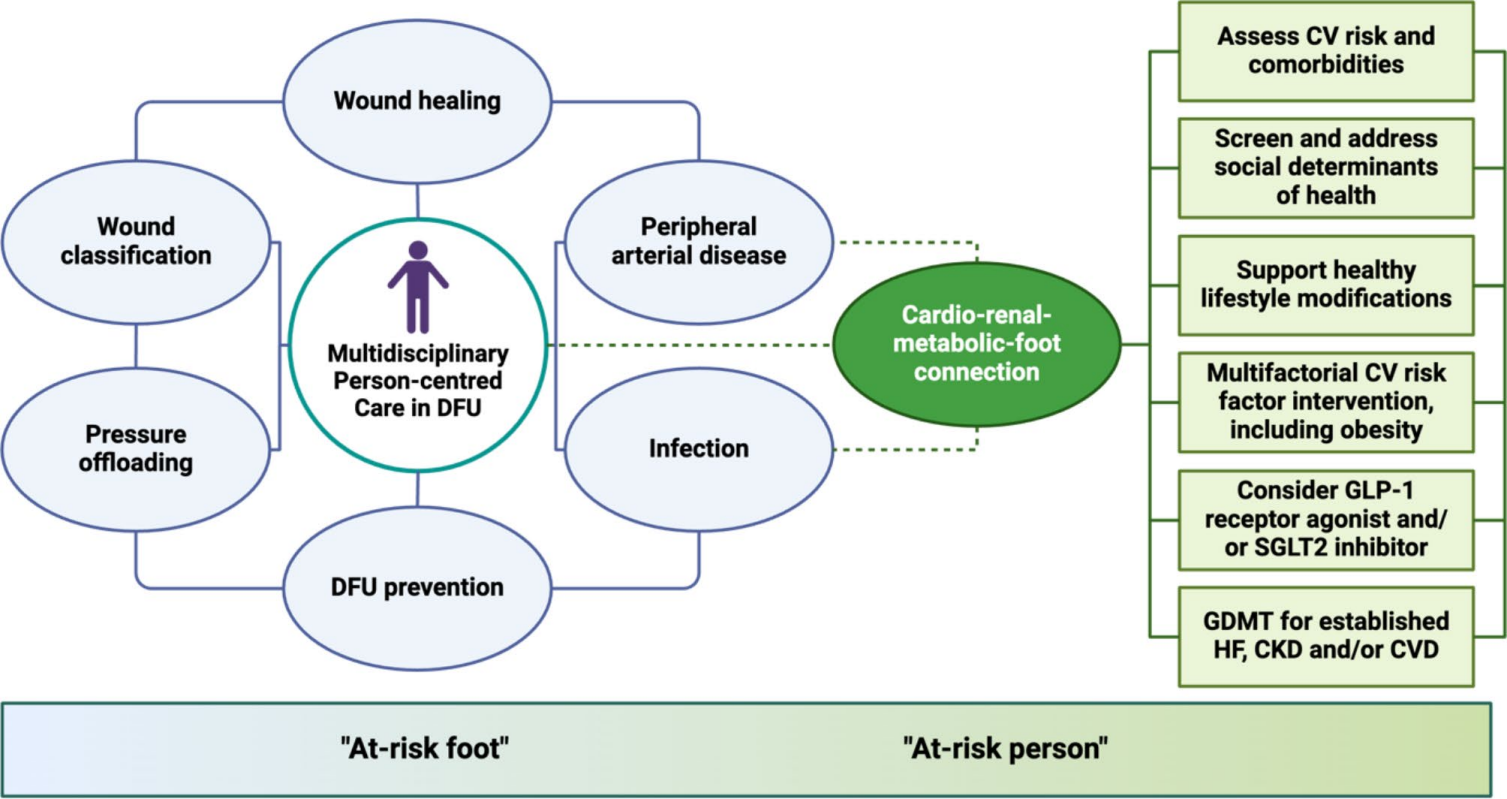
An example of a cardio-renal-metabolic-foot model of care in people with DFU. The current standard of care for DFU addresses wound classification, pressure offloading, infection, peripheral arterial circulation, wound debridement and dressings, and wound treatments for non-healing ulcers. However, there is a need for new emphasis on the cardio-renal-metabolic-foot connection, given that CVD, HF and CKD are intractable issues for people with DFU. The model includes multifactorial CV risk factor intervention, the use of medications with CV and renal benefits, and further emphasises the need to address the social determinants of health. The goal is to manage not only the DFU (i.e., the “at-risk foot”), but to also prevent and manage associated cardiac and renal complications (i.e., the “at-risk person”). *CKD* chronic kidney disease, *CV* cardiovascular, *CVD* cardiovascular disease, *DFU* diabetes-related foot ulceration, *GDMT* guideline-directed medical therapy, *GLP-1* glucagon-like peptide-1, *HF* heart failure, *SGLT2* sodium-glucose cotransporter 2. *Created with BioRender.com*

The care of people with DFU is challenging, requiring attention to several inter-related comorbidities. To reduce episodic and fragmented care, people with DFU should be treated by a dedicated multidisciplinary team, with shared-decision making being imperative [10, 105]. Early, high-quality, and holistic team-based care improves healing of DFU, reduces lower extremity amputations and hospitalisations, and is cost-effective [10, 106, 107]. The current standard of care includes wound classification/staging, identifying and addressing infection, assessment of peripheral arterial circulation with revascularisation by endovascular and/or open approaches where appropriate, pressure offloading, wound debridement and dressings, and consideration of wound treatments for non-healing ulcers [10, 105]. Nonetheless, the goal should be to also manage the person beyond the DFU (“at-risk foot”) by actively identifying and managing associated cardiac, renal and metabolic issues (“at-risk person”) [10, 40]. In people with DFU, concomitant CV and renal comorbidities are highly prevalent and are associated with significantly increased risk of mortality [34]. However, there is an expanding number of therapies that can reduce the risk of progression, and improve the prognosis of, atherosclerotic CVD, heart failure and CKD. Thus, the integration of novel models of care that addresses the multi-directional cardio-renal-metabolic-foot connection may be important. The proposed model (Fig. 2) would target the “at-risk foot” and the “at-risk person”.

### Cardiovascular and renal risk-reducing therapies

**Fig. 3:**
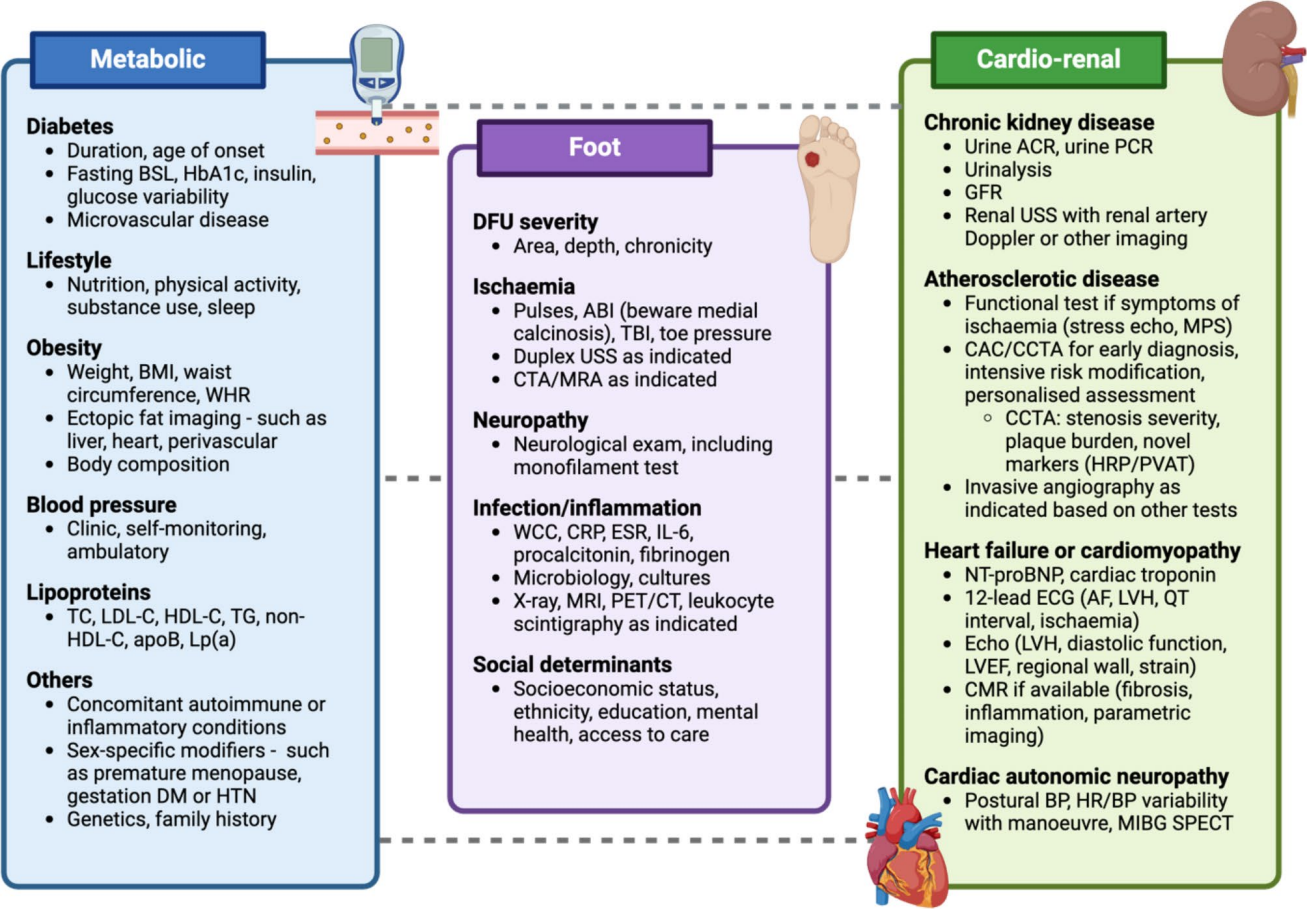
Proposed clinical cardiovascular risk assessment strategy in people with DFU. The authors propose an example CV risk assessment strategy that considers several clinical factors and biomarkers, including DFU-related factors, to facilitate risk stratification and to guide the implementation of strategies to reduce the risk of future CV events. Treating clinicians should apply clinical judgement and personalised decision-making in the CV risk assessment strategy. *ABI* ankle-brachial index, *ACR* albumin-creatinine ratio, *AF* atrial fibrillation, *apoB* apolipoprotein B, *BMI* body mass index, *BP* blood pressure, *BSL* blood sugar level, *CAC* coronary artery calcium, *CCTA* coronary computed tomography angiography, *CMR* cardiac magnetic resonance, *CRP* C-reactive protein, *CT* computed tomography, *CTA* computed tomography angiography, *DFU* diabetes-related foot ulceration, *DM* diabetes mellitus, *ECG* electrocardiogram, *echo* echocardiography, *ESR* erythrocyte sedimentation rate, *GFR* glomerular filtration rate, *HbA1c* glycated haemoglobin, *HDL-C* high-density lipoprotein cholesterol, *HR* heart rate, *HRP* highrisk plaque, *IL-6* interleukin-6, *LDL-C* low-density lipoprotein cholesterol, *Lp(a)* lipoprotein(a), *LVH* left ventricular hypertrophy, *MIBG* iodine-123 metaiodobenzylguanidine, *MPS* myocardial perfusion scan, *MRA* magnetic resonance angiography, *MRI* magnetic resonance imaging, *NT-proBNP* N-terminal pro b-type natriuretic peptide, *PCR* protein-creatinine ratio, *PET* positron emission tomography, *PVAT* peri-vascular adipose tissue, *SPECT* single-photon emission computed tomography, *TBI* toe-brachial index, *TC* total cholesterol, *TG* triglyceride, *TTE* transthoracic echocardiography, *USS* ultrasound scan, *WCC* white cell count, *WHR* waist-to-hip ratio. *Created with BioRender.com*

People with DFU are generally at high or very high CV risk given that DFU is associated with other complications of diabetes (e.g., neuropathy, PAD and/or CKD) and a greater burden of CV risk factors [10, 38, 105]. CV risk assessment should be personalised and consider several clinical factors and biomarkers in people with DFU, including DFU-related factors, to guide CV risk reduction strategies (Fig. 3). Along with lifestyle changes such as exercise, healthy eating patterns, smoking cessation and weight loss where appropriate, CV and renal risk-reducing therapies are recommended (Table 2) in people with DFU [90, 91, 108–111]. Given the scarcity of CV and renal outcome trials evaluating risk-reducing therapies in people with DFU, evidence is often extrapolated from studies that included people with type 2 diabetes. Recent guidelines for the prevention and management of CVD or CKD in people with diabetes should be followed and adapted to local policies, practices, and resources [90, 91, 108–110]. People with diabetes derive greater absolute benefits from risk-reducing strategies, such as lipid-lowering, owing to their higher absolute CV risk compared with people without diabetes [90, 112]. It is likely that people with DFU may benefit more, owing to their even higher risk. Crucially, implementation of an intensive CV risk factor screening program and use of risk-reducing therapies, where statins were recommended for all, was associated with a reduction in 5-year mortality from 48 to 27% in an observational study of people with DFU [113]. 

**Table 2:** Cardiovascular and renal risk-reducing and prognostic pharmacotherapies to consider in people with DFU [90, 91, 108–111]

Drug mechanism	Medications (or class)	Treatment goals informed by recent international guidelines (ADA, AHA, ESC and KDIGO)
Anti-thrombotic	Aspirin Rivaroxaban	Aspirin (or clopidogrel if aspirin intolerant) is recommended in people with established CVD (e.g., PAD) [class I level A] The benefits of aspirin in people without overt CVD needs to be weighed against bleeding risks [class IIb level A]; in people with DFU, the benefits of aspirin are likely to outweigh risks. Rivaroxaban (at very low dose) added to low dose aspirin reduces CV risk in people with chronic coronary syndrome or symptomatic PAD [class IIb level A]
Glucose-lowering	GLP-1 agonist SGLT2 inhibitor	Treatment with these agents reduces CV risk independent of glycaemic control GLP-1 agonists^a^ and SGLT2 inhibitors^b^ reduce CV risk in people with T2D and CVD or multiple CV risk factors [class I level A] SGLT2 inhibitors^b^ reduce HF hospitalisation or CV death in people with T2D and HF irrespective of LVEF [class I level A] SGLT2 inhibitors^c^ reduce CV and kidney failure risk in people with T2D and CKD [class I level A] Consider GLP-1 agonist in people with T2D and CKD who have not achieved glycaemic targets despite metformin and SGLT2 inhibitor treatment [level I grade B] A HbA1c goal < 7% is recommended for most nonpregnant adults; glycaemic targets should be individualised and hypoglycaemia avoided
Cholesterol-lowering	Statin (high intensity) Ezetimibe Bempedoic acid PCSK9-directed therapy	Treatment with these agents reduces the level of apoB-containing lipoproteins and LDL-C, and consequently, reduces CV risk Statins are first-line treatment and should be commenced in people with diabetes and established CVD or who are age ≥ 40 years [class I level A] LDL-C is the primary target; the goal in very-high risk people is < 55 mg/dL (1.4 mmol/L) and in high risk people is < 70 mg/dL (1.8 mmol/L)^d^ Combination therapy is often required to attain goals
Triglyceride-lowering	Icosapent ethyl	Icosapent ethyl added to statin in people with diabetes, additional CV risk factors (or established CVD) and hypertriglyceridaemia (135–499 mg/dL or 1.5–5.6 mmol/L) reduces CV risk [class IIb level B]
Anti-inflammatory	Colchicine	Colchicine (at low dose) may be considered for secondary prevention of CVD, particularly if other risk factors are insufficiently controlled or there are recurrent CV events despite optimal therapy
Neuro-hormonal blockade	ACE inhibitor, ARB or ARNI β-blocker Steroidal MRA Non-steroidal MRA	ACE inhibitor *or* ARB with a CCB or thiazide/thiazide-like diuretic is recommended for the treatment of hypertension [class I level A] A BP goal ≤ 130/80 mmHg is recommended for most nonpregnant adults; BP goals should also be individualised ARNI *or* ACE inhibitor, beta-blocker, steroidal MRA and SGLT2 inhibitor combination therapy as soon as possible reduces HF hospitalisation and death in people with HF with reduced LVEF [class I level A] ACE inhibitor *or* ARB reduces kidney failure risk in people with T2D and CKD [class I level A] Finerenone (a non-steroidal MRA) added to ACE inhibitor *or* ARB reduces HF hospitalisation and kidney failure risk in people with T2D and CKD [class I level A]

*ACE* angiotensin-converting enzyme inhibitor, *ADA* American Diabetes Association, *AHA* American Heart Association, *apoB* apolipoproteinB, *ARB* angiotensin II receptor blockers, *ARNI* angiotensin receptor/neprilysin inhibitors, *BP* blood pressure, *CCB* calcium-channel blocker, *CKD* chronic kidney disease, *CV* cardiovascular, *CVD* cardiovascular disease, *ESC* European Society of Cardiology, *GLP-1* glucagon-like peptide-1, *HF* heart failure, *KDIGO* Kidney Disease Improving Global Outcomes, *LDL-C* low-density lipoprotein cholesterol, *LVEF* left ventricular ejection fraction, *MRA* mineralocorticoid receptor antagonist, *PAD* peripheral arterial disease, *PCSK9* proprotein convertase subtilisin/kexin type 9, *SGLT2* sodium-glucose cotransporter 2, *T2D* type 2 diabetes

^a^Dulaglutide, efpeglenatide, liraglutide and semaglutide

^b^Canagliflozin, dapagliflozin, empagliflozin and sotagliflozin

^c^Canagliflozin, dapagliflozin and empagliflozin

^d^Several methods of risk stratification exist. The ESC 2019 guidelines define very-high risk as: diabetes with established CVD, target organ damage (e.g., microvascular disease) or three or more other risk factors or type 1 diabetes duration of > 20 years. High risk is defined as: diabetes duration of > 10 years or diabetes with one or more other risk factors

Glycaemia should be addressed as part of multifactorial risk factor reduction [114]. However, people with DFU often have a long duration of diabetes and advanced microvascular or macrovascular complications underlying the DFU. Glycaemic targets should therefore be individualised, taking into consideration factors such as comorbidities, frailty, life expectancy, risk of hypoglycaemia and patient preferences [114]. The effect of glycaemic control on DFU healing is not known, as no randomised trials have been performed to address this question and data from observational studies are conflicting [115–118]. However, glycaemic management often remains necessary, as poor glycaemic control has been associated with serious infections and recurrence of DFU [119, 120]. Optimising glycaemia can reduce the progression of microvascular disease and is associated with a reduced risk of lower extremity amputation in people with diabetes [114, 121]. Further studies are required to assess whether glycaemic variability associates with DFU outcomes and CV outcomes in people with DFU [122–124]. Glucose-lowering therapies with proven CV and renal prognostic benefits independent of glycaemia in people with type 2 diabetes such as glucagon-like peptide-1 (GLP-1) agonists and sodium-glucose cotransporter 2 (SGLT2) inhibitors should be prioritised and their use in combination be considered [3, 125, 126]. Whilst there are no randomised trials evaluating CV benefits of GLP-1 agonists and SGLT2 inhibitors in combination, observational studies have suggested that the combination is associated with a lower risk of CV events and serious renal events compared with either drug class alone [127]. GLP-1 agonists and SGLT2 inhibitors are not approved by the US Food and Drug Administration (FDA) for the treatment of people with type 1 diabetes, a cohort in whom trial data to guide CV prevention is lacking [91, 128, 129]. 

However, uncertainty remains as to whether it is safe to prescribe SGLT2 inhibitors in people with DFU [130]. Recent international guidelines recommend that SGLT2 inhibitors should not be initiated in drug naïve people with a DFU and that temporary discontinuation should be considered in people already on an SGLT2 inhibitor until the affected foot is healed [130]. Although canagliflozin was associated with an increased risk of lower extremity amputations in the CANVAS trial, this was not observed in trials of other SGLT2 inhibitors, in the CREDENCE trial of canagliflozin, or in a meta-analysis, perhaps owing to increased awareness, preventive measures, and/or the avoidance of including people at high risk of amputations in trials [126, 131, 132]. A pharmacovigilance analysis of the US FDA adverse event Reporting System found that canagliflozin, but not dapagliflozin or empagliflozin, was associated with an increased risk of amputations [133]. The US FDA implemented a Boxed Warning regarding risk of amputation with canagliflozin, but this was later removed based on review of new clinical trial data. Whilst some caution the use of SGLT2 inhibitors in people with active DFU, we consider that an individualised person centred-approach be adopted in people with heart failure and/or CKD where the uncertain, and possibly low risk, of exacerbating the DFU is balanced with the benefits of SGLT2 inhibitor use [130]. If an SGLT2 inhibitor is prescribed, adherence to wound care and close monitoring of the DFU by both the clinician and patient is required. As volume depletion has been hypothesised to contribute to the increased risk of amputation associated with canagliflozin, careful titration of concomitant diuretics and measures to avoid hypovolemia would seem appropriate [134, 135]. Patients should also be instructed to withhold SGLT2 inhibitors during acute illness (e.g., infection, fasting, diarrhea or vomiting) to reduce the risk of hypovolemia and ketosis. Conversely, a meta-analysis of observational studies showed that GLP-1 agonists may be associated with a reduced risk of amputations [136]. A post-hoc analysis of a CV outcome trial examining liraglutide in people with type 2 diabetes at high CV risk found that liraglutide did not increase the risk of DFU events and was associated with a significantly lower risk of DFU-related amputations compared with placebo [137]. 

### Cardiovascular disease, heart failure and chronic kidney disease

In people with DFU and established atherosclerotic CVD (e.g., PAD), the use of aspirin or P2Y_12_ inhibitors with high-intensity statin is indicated [90]. The addition of ezetimibe, bempedoic acid and/or therapies directed at proprotein convertase subtilisin/kexin type 9 (e.g., monoclonal antibodies or RNA-based therapies) should be guided by low-density lipoprotein cholesterol goals and/or thresholds for treatment [90, 111, 112, 138–141]. In people with diabetes and CAD or PAD without high bleeding risk, the addition of very low-dose rivaroxaban to low-dose aspirin is recommended to address residual thrombotic risk [142–144]. Moreover, icosapent ethyl may be considered for people with diabetes, additional CV risk factors, and residual hypertriglyceridaemia (135–499 mg/dL) despite statin therapy [111, 145]. Although lipoprotein(a) measurement remains uncommon, there is some evidence that higher levels may be associated with an increased risk of developing DFU [146]. In people with elevated lipoprotein(a), intensive risk factor management for CV prevention should be prioritised in the absence of specific lipoprotein(a)-lowering therapies [147]. 

In the setting of heart failure with reduced ejection fraction, guideline-directed medical therapy is indicated with a focus on the 4 pillars of β-blockers, angiotensin receptor/neprilysin inhibitors, mineralocorticoid receptor antagonists and SGLT2 inhibitors [90]. In heart failure with mildly reduced or preserved ejection fraction, SGLT2 inhibitors are recommended [90]. Moreover, kidney-protective therapies with CV benefits such as angiotensin-converting enzyme inhibitors or angiotensin II receptor blockers, SGLT2 inhibitors, finerenone (non-steroidal mineralocorticoid receptor antagonist) and semaglutide (GLP-1 agonist), should be considered in people with DFU and CKD [132, 148–152]. The combination of these therapies has been coined as the 4 pillars of guideline-directed medical therapy for type 2 diabetes and CKD, but questions remain regarding the order of initiation and how rapidly these medications should be initiated [153]. The complex interplay between metabolic risk factors, CKD and CVD and the rapidly transforming therapeutic landscape highlights unique management considerations in people with DFU. In diabetes, albuminuria is a major amplifier of atherosclerotic CVD and heart failure risk. Importantly, trials of SGLT2 inhibitors, finerenone and semaglutide have demonstrated that these therapies not only reduced the incidence of renal outcomes, but also major adverse CV events compared with placebo in people with type 2 diabetes and CKD [132, 148, 149, 152, 154]. Moreover, SGLT2 inhibitors and finerenone can reduced the risk of heart failure in people with type 2 diabetes and CKD [132, 148, 149, 154]. These classes of medication have differing mechanisms of action that target interrelated haemodynamic, metabolic, inflammatory and fibrotic pathways, thereby addressing multiple aspects of the proposed cardio-renal-metabolic-foot connection in people diabetes [3]. 

### Social determinants of health

The social determinants of health should be systematically screened using validated tools and addressed using local resources, given their impact on DFU, CV and renal outcomes [3, 155]. Limited or delayed access to preventive or specialist care contributes to late-stage DFU presentation and worse outcomes [40]. Widely disparate health outcomes in terms of amputation and mortality rates have been observed based on ethnicity, geographic location, and socioeconomic status [40, 156–158]. The psychosocial impact of DFU on quality of life and self-care, particularly due to disability, could also impact on CV and renal outcomes. Crucially, people living with DFU often report poor quality of life, depression and anxiety [159–161]. Furthermore, financial strain should be considered, given that personal costs associated with DFU can be substantial depending on the health care system [162]. Health services should strive for equitable access to specialist care and pharmacotherapies, and increased funding for DFU care initiatives, infrastructure, education and research. Hopefully, the gap between current resource allocation and the significant burden of DFU and CVD can be bridged [163]. 

## Future research considerations

**Fig. 4:**
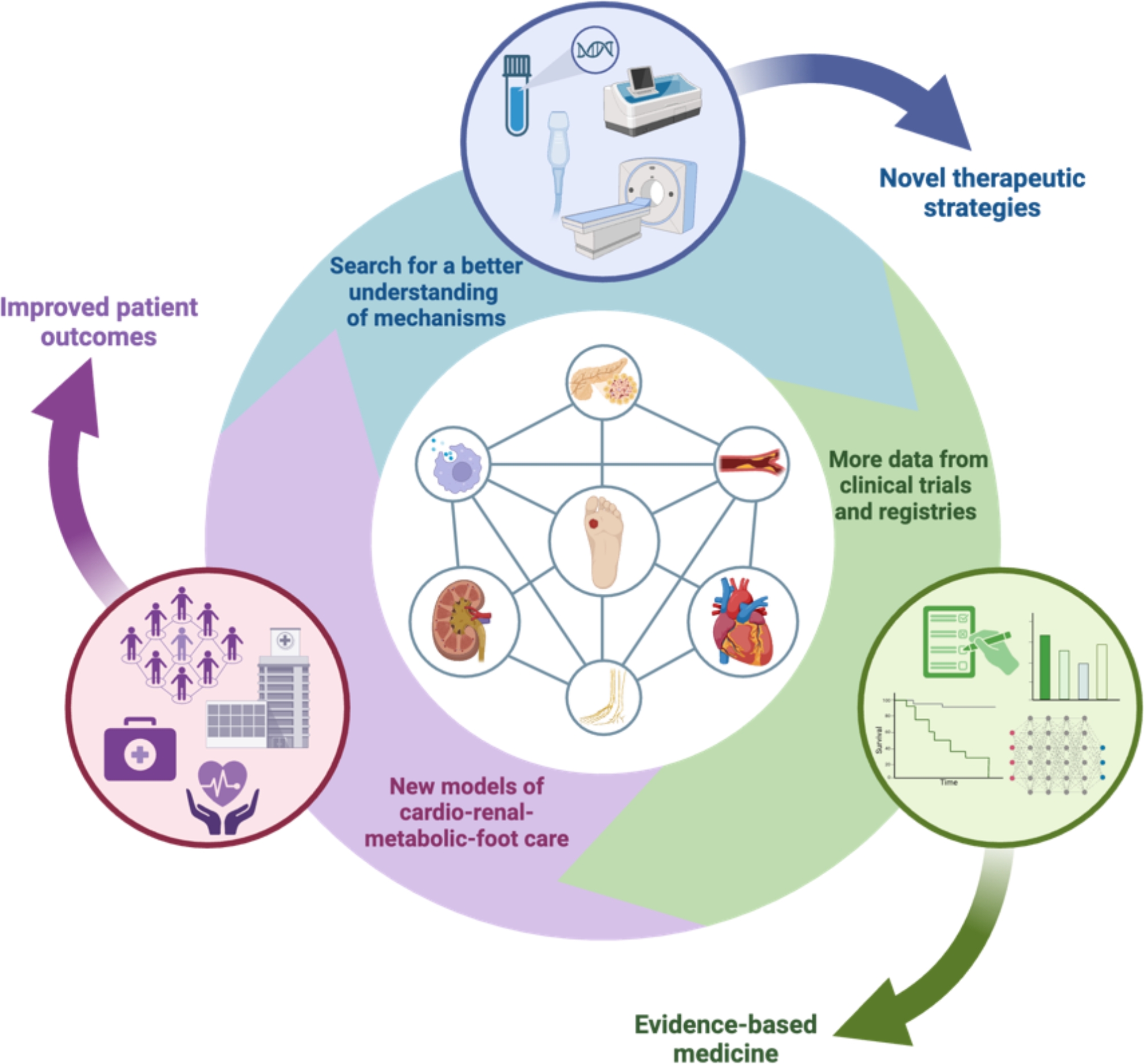
Research considerations in the cardiovascular care of people with DFU. There is a critical need for (1) a better understanding of mechanisms connecting DFU with CV and renal disease, perhaps guided by novel biomarkers, multi-omics, advanced multi-modality cardiac imaging and artificial intelligence to facilitate current treatments and the development of novel therapeutic strategies, (2) more data from clinical trials, registries and biobanks to inform clinical guidelines and evidence-based medicine, and (3) health system-wide integration of structured models of care with a contemporary emphasis on cardio-renal-metabolic-foot health to improve patient outcomes. *DFU* diabetes-related foot ulceration. *Created with BioRender.com*

Numerous knowledge gaps drive the need for focused research in key areas pertaining to DFU and CV care (Fig. 4). As previously highlighted, there is a need for a better understanding of underlying disease mechanisms connecting DFU with CVD and CKD. If the inflammatory “milieu” associated with DFU is a driver of atherosclerosis, cardiac remodelling, and CKD, then it may be postulated that anti-inflammatory therapies have a preventive role. Such a strategy may unfortunately be hampered by infectious complications secondary to immune system suppression [67, 164]. Manipulation of the immune system using immunomodulatory therapies is an attractive concept, but ongoing research is needed [60, 67]. Colchicine is an anti-inflammatory that can reduce CV events in secondary prevention cohorts, but its use in people with CKD is cautioned; thus, its applicability in people with DFU may be reduced [165]. Whether people with DFU derive greater CV benefits from anti-inflammatory therapies due to heightened chronic inflammation remains unknown. Several therapeutic targets to lessen inflammation in diabetes have been studied and shared targets between DFU and diabetic cardiomyopathy may exist [166, 167]. Here, the combined analysis of metabolomics, lipidomics and proteomics offers new insights to facilitate precision medicine [167]. Bioinformatics analyses have suggested that fenofibrate could be a therapeutic agent for diabetic cardiomyopathy and DFU, as the *PPARG* gene is implicated in both [167]. 

Approaches which promote resolution of inflammation may also reduce CV and renal risk [164]. In the context of DFU, this raises the question as to whether earlier treatment and/or therapies to accelerate wound healing and reduce inflammation may improve prognosis [168]. Wound healing depends on a tightly regulated process involving fibroblasts, endothelial cells, phagocytes, platelets, and growth factors [168]. Direct evidence linking well-established CV risk factors such as dyslipidaemia and hypertension to healing of DFU has not been shown [10]. Interestingly, heart failure has been associated with delayed healing of DFU, which could suggest a bidirectional relationship between DFU and the heart or that confounders such as frailty may be contributing [169, 170]. Whether SGLT2 inhibitors or GLP-1 agonists improve healing of DFU independent of lower extremity perfusion requires investigation [10]. In clinical trials are novel therapies for wound healing that target tissue repair, infection, and/or blood supply and this has been reviewed elsewhere [168]. 

Furthermore, data from high-quality international registries, databases, biobanks, and clinical trials will enable evidence-based medicine and reduce the disparity of care. Studies in DFU should evaluate differences in CV and renal outcomes according to sex and diabetes type (e.g., type 1 versus type 2). CV risk prediction models for DFU should also be considered to assist with shared decision-making; these models would ideally incorporate diabetes and DFU-specific factors and the socioeconomic determinants of health. A comprehensive assessment of the ulcer(s) is essential to identify its size (e.g., area and depth), severity, infections and chronicity, and therefore potential impact on the CV system, given that a deep and infected ulcer may increase the risk of future CV events. Here, medical technology can provide more precise measurements and healing trends [171]. Artificial intelligence has already begun to revolutionise healthcare and has the potential to offer novel strategies to risk-stratify people with DFU [172]. Machine or deep learning algorithms may play a role in various aspects of DFU care, including screening, lesion segmentation, assessment of severity, monitoring, cardiac imaging, and prediction of adverse CV or renal sequelae [173–175]. 

## Conclusion

People with DFU have a high prevalence of CV risk factors and comorbidities including CKD, resulting in an excessively high risk of mortality from CVD that exceeds many aggressive forms of cancer. Shared pathophysiological mechanisms connecting DFU with CVD and CKD suggests that a multi-directional cardio-renal-metabolic-foot connect may exist. The expanding number of therapies that have beneficial metabolic, renal and CV effects offers promise in improving health outcomes for people with DFU. However, more research is needed to enhance our understanding of underlying pathology, screening, prevention, and optimal management of cardio-renal-metabolic-foot health in people with DFU. Here, cardiac imaging could play a crucial role in improving our understanding of disease mechanisms and in personalising risk assessment and management. Ultimately, there is a need for (1) a better understanding of mechanisms connecting DFU with CVD and CKD to inform novel therapeutic strategies, (2) more data from clinical trials, registries, and biobanks to inform clinical guidelines, and (3) health system-wide integration of novel models of care, with a new focus on the cardio-renal-metabolic-foot connection.

## Data Availability

No datasets were generated or analysed during the current study.

## Abbreviations

CAD: Coronary artery disease
CCTA: Coronary computed tomography angiography
CKD: Chronic kidney disease
CV: Cardiovascular
CVD: Cardiovascular disease
DFU: Diabetes-related foot ulceration
GLP-1: Glucagon-like peptide-1
PAD: Peripheral arterial disease
SGLT2: Sodium-glucose cotransporter 2
